# Generation of Bladder Urothelium from Human Pluripotent Stem Cells under Chemically Defined Serum- and Feeder-Free System

**DOI:** 10.3390/ijms15057139

**Published:** 2014-04-25

**Authors:** Minyong Kang, Hyeon Hoe Kim, Yong-Mahn Han

**Affiliations:** 1 Graduate School of Medical Science and Engineering, KAIST, 291 Daehak-ro, Yuseong-gu, Daejeon 305-701, Korea; E-Mail: kmy5226@kaist.ac.kr; 2 Department of Urology, Seoul National University Hospital, 101 Daehak-ro, Jongno-gu, Seoul 110-744, Korea; E-Mail: hhkim@snu.ac.kr; 3 Department of Biological Sciences, KAIST, 291 Daehak-ro, Yuseong-gu, Daejeon 305-701, Korea

**Keywords:** pluripotent stem cells, cell differentiation, definitive endoderm, urinary bladder, urothelium

## Abstract

Human stem cells are promising sources for bladder regeneration. Among several possible sources, pluripotent stem cells are the most fascinating because they can differentiate into any cell type, and proliferate limitlessly *in vitro*. Here, we developed a protocol for differentiation of human pluripotent stem cells (hPSCs) into bladder urothelial cells (BUCs) under a chemically defined culture system. We first differentiated hPSCs into definitive endoderm (DE), and further specified DE cells into BUCs by treating retinoic acid under a keratinocyte-specific serum free medium. hPSC-derived DE cells showed significantly expressed DE-specific genes, but did not express mesodermal or ectodermal genes. After DE cells were specified into BUCs, they notably expressed urothelium-specific genes such as *UPIb*, *UPII*, *UPIIIa*, *P63* and *CK7*. Immunocytochemistry showed that BUCs expressed UPII, CK8/18 and P63 as well as tight junction molecules, E-CADHERIN and ZO-1. Additionally, hPSCs-derived BUCs exhibited low permeability in a FITC-dextran permeability assay, indicating BUCs possessed the functional units of barrier on their surfaces. However, BUCs did not express the marker genes of other endodermal lineage cells (intestine and liver) as well as mesodermal or ectodermal lineage cells. In summary, we sequentially differentiated hPSCs into DE and BUCs in a serum- and feeder-free condition. Our differentiation protocol will be useful for producing cells for bladder regeneration and studying normal and pathological development of the human bladder urothelium *in vitro*.

## 1. Introduction

The urinary bladder is essential for storage and voiding of urine from the body. Bladder cancer, congenital anomalies, spinal cord injury, and chronic inflammation can irreversibly damage the bladder [1], and most patients suffering from these problems need surgery for bladder reconstruction [2]. Reconstruction of bladder tissue and finding suitable sources for bladder regeneration are major challenges in the field of regenerative medicine [3]. At present, gastrointestinal substitution is commonly employed for bladder reconstruction [4]. However, complications associated with this approach due to the functional differences between the bladder and intestine tissues include recurrent infections, bladder stones, metabolic disturbances, and carcinomas [5]. Thus, an artificial or bioengineered bladder would be the ideal materials for regeneration of the urinary bladder, both of which would need to be lined with a stratified epithelium called the urothelium [6]. The urothelium lines the inner surface of the urinary bladder and provides a barrier against pathogens, toxins and waste products in the urine [7].

Despite the urgent need for bladder tissue engineering, suitable sources for human urothelium have been difficult to obtain. Maintenance and expansion of primary bladder cells is not feasible because prolonged culture of primary cells gives rise to cellular senescence [8]. Instead, several types of stem cells are receiving much attention for their potential in bladder regeneration [9]. Over the last decade, adult stem cells have been discovered in adult tissues such as bone marrow, amniotic-fluid, and adipose tissues [3]. Although adult stem cells are very useful for autologous cell therapies without immune rejection, their clinical utility is still limited due to ethical problems in isolation from human donors, their low isolation efficiency, and imperfect culture systems for expansion *in vitro* [10,11]. In addition, the usefulness of adult stem cells for tissue-replacement therapy is questionable because many of the beneficial effects of these cells have been attributed to their paracrine activities, not their direct differentiation into the relevant cell types [12]. Alternatively, human pluripotent stem cells (hPSCs) such as human embryonic stem cells (hESCs) and induced pluripotent stem cells (hiPSCs) can proliferate indefinitely and differentiate into any of the cell types in the body [13]. For clinical use, hPSCs should be differentiated into the desired cell type to reduce the risk of teratoma formation when the cells are transplanted into the patient [14]. Several studies of *in vitro* urothelial differentiation have been reported using mouse embryonic stem cells (mESCs) [15–17]. Recently, hiPSCs derived from urinary tract cells were shown to differentiate into urothelium by co-culturing with bladder stromal cells or culturing in a conditioned medium [18]. However, co-culturing or animal serum-containing system is limited for cell therapy using hPSC-derived cells because unknown factors contained in this system can trigger unexpected side effects. Thus, differentiation of hPSCs into urothelium using a chemically defined system will be much more beneficial in clinical applications for bladder regeneration. In this study, we developed a protocol for differentiation of hPSCs into bladder urothelial cells (BUCs) in a serum- and feeder-free system using a developmental stage-dependent approach.

## 2. Results and Discussion

### 2.1. Results

**Figure 1.**
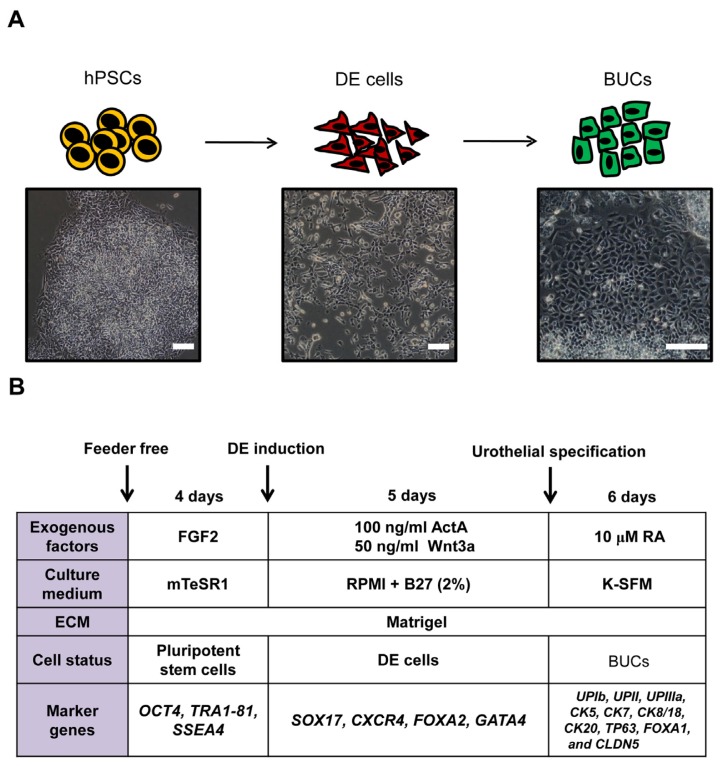
Schematic protocol for differentiation of human pluripotent stem cells (hPSCs) into bladder urothelial cells (BUCs). (**A**) Illustration and phase contrast images show morphologic changes of human pluripotent stem cells during differentiation into definitive endoderm (DE) and BUCs. Scale bars = 200 μm; (**B**) Detailed protocol for BUCs differentiation from hPSCs.

Schematic illustration and overall protocol for differentiation of hPSCs into BUCs are shown in Figure 1. Briefly, we initially differentiated hPSCs into DE, and further specified DE cells into BUCs. We hypothesized that DE induction is prerequisite for efficient derivation of BUCs from hPSCs as a developmental stage-dependent approach. As shown in Figure 1A, the morphology of hPSCs gradually changed into rice-grain appearance as the monolayers of epitheloid cells after BUCs induction in phase contrast images, typical in cultured urothelial cells [19].

#### 2.1.1. Induction of Definitive Endoderm from hESCs

**Figure 2.**
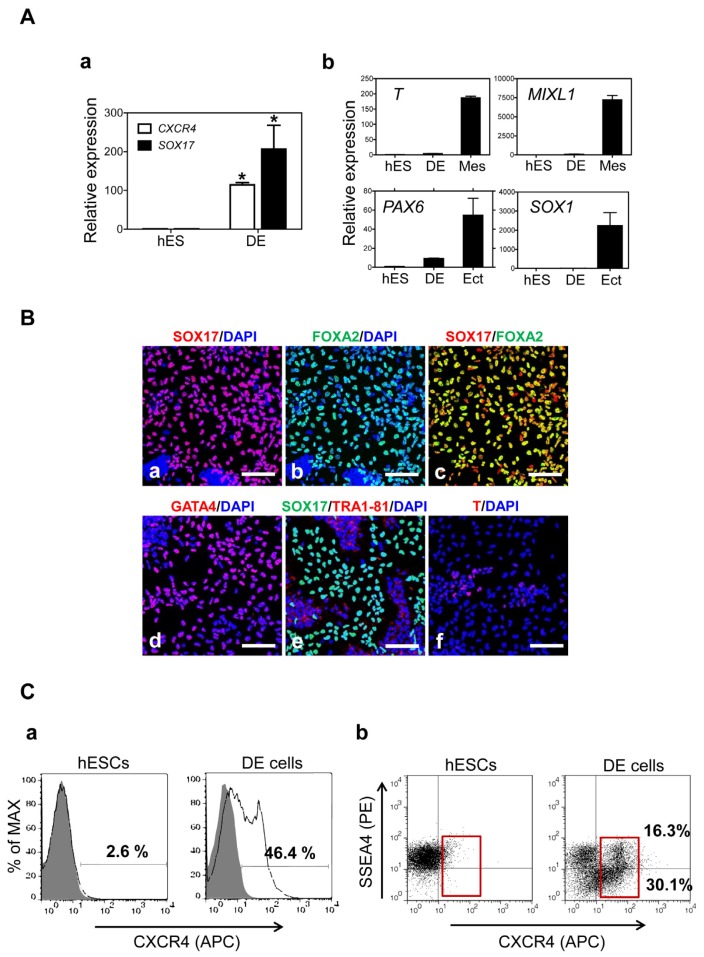
Induction of hESCs into DE cells. (**A**) (**a**) DE induction was confirmed by transcriptional expression of the DE marker genes *CXCR4* and *SOX17*. Relative expression of marker genes was normalized to *GAPDH* and the fold changes were shown as the mean ± SEM (*n* = 3, * *p* < 0.05); (**b**) Heterogeneous differentiation into other germ layers in DE induction conditions, including mesoderm and ectoderm, was evaluated by expression of mesodermal (*T* and *MIXL1*) and ectodermal genes (*PAX6* and *SOX1*). hESCs were used as a negative control. Mesoderm (Mes) and ectodermal (Ect) cells were used as positive controls; (**B**) Immunocytochemistry analysis of expression of (**a**–**d**) DE markers (SOX17, FOXA2 and GATA4); an (**e**) undifferentiated marker (TRA1-81); and a (**f**) mesodermal marker (T) in the DE cells. Scale bar = 200 μm; (**C**) Flow cytometry analysis of (**a**) CXCR4 expression as a DE-specific marker; and (**b**) co-expression of CXCR4 and SSEA4 in hESCs and hESC-derived DE cells. Red boxes indicate the region of CXCR4-positive cells, with or without SSEA-4 expression.

When hESCs were cultured in DE induction medium supplemented with Activin A and Wnt3a (AW) for five days, transcriptional expression of the DE marker genes *CXCR4* and *SOX17* was markedly enhanced compared to undifferentiated hESCs (Figure 2A(a)). However, expression of other lineage marker genes such as *T* or *MIXL1* (mesoderm) and *PAX6* or *SOX1* (ectoderm) was low or absent in DE cells (Figure 2A(b)). Additionally, immunocytochemistry showed that key DE markers SOX17, FOXA2 and GATA4 were clearly detected in the nuclei of hESC-derived DE cells (Figure 2B(a,b,d)). We confirmed that SOX17 and FOXA2 were co-expressed in the nuclei of DE cells (Figure 2B(c)). As shown in Figure 2B(e), SOX17-positive cells were clearly distinguished from cells expressing the pluripotent marker TRA1-81. A small subpopulation of hESC-derivatives was positive for T, consistent with the transcriptional expression of *T* (Figure 2B(f)). Flow cytometry analysis showed that approximately 46% of hESC-derivatives after DE induction were CXCR4-positive cells compared to undifferentiated hESCs (Figure 2C(a)). Of this CXCR4-positive population, approximately 16% of CXCR4-positive cells co-expressed the pluripotency marker SSEA4 (Figure 2C(b)), which would indicate cells at an intermediate stage of DE induction.

#### 2.1.2. Specification of hESC-Derived DE Cells into BUCs

**Figure 3.**
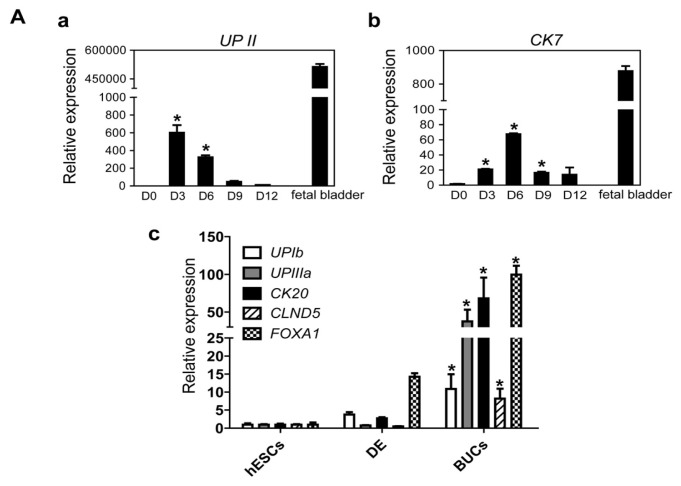

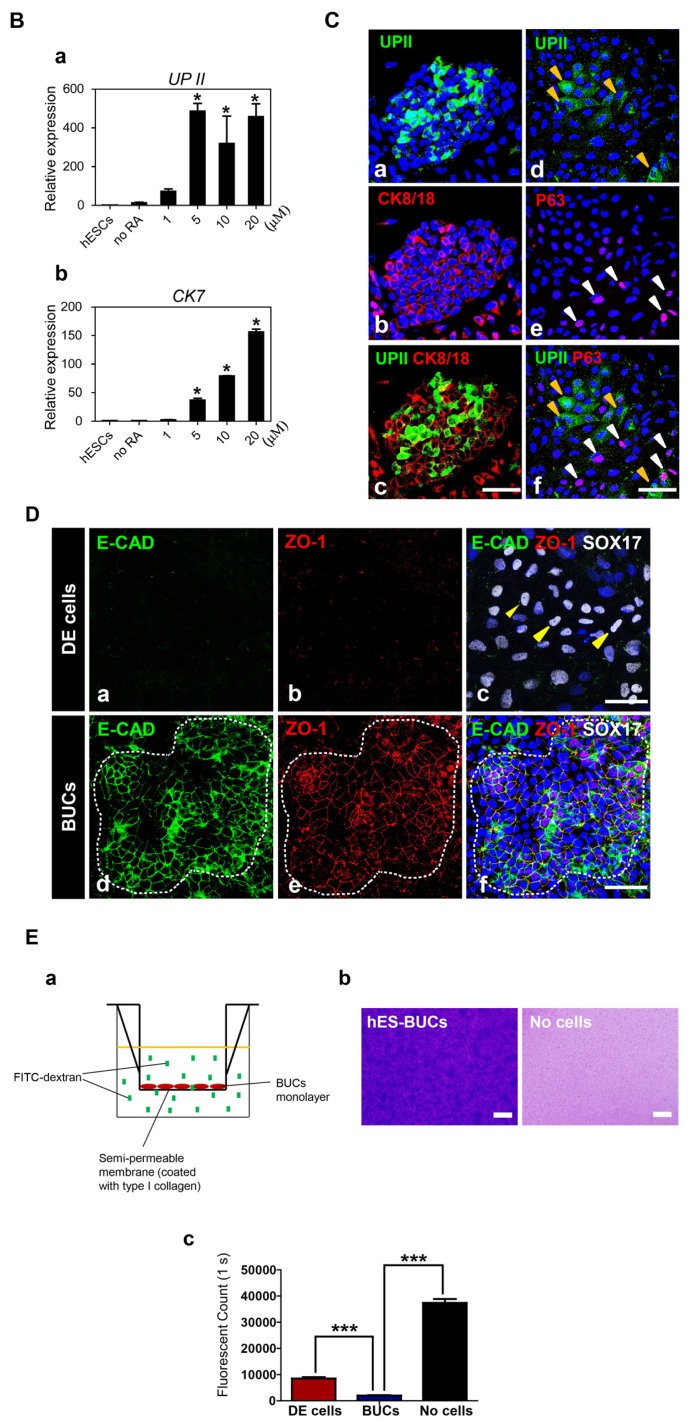

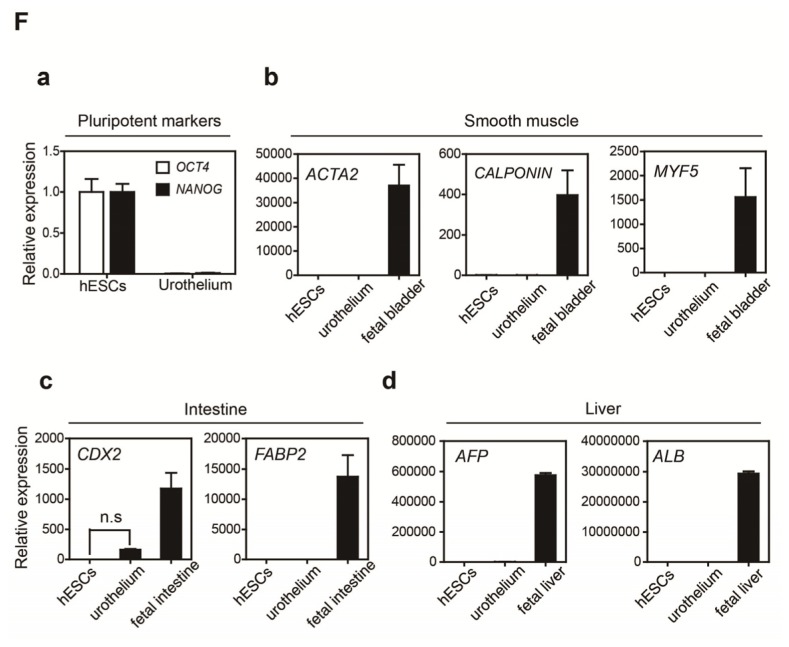
Specification of hESC-derived DE cells into BUCs. (**A**) Optimal timing for induction of BUCs from DE cells was determined by transcriptional expression level of the key marker genes (**a**) *UPII (UROPLAKINII)* and (**b**) *CK7 (CYTOKERATIN 7)*, compared to undifferentiated hESCs as a negative control. Human fetal bladder cDNA was used as a positive control (designated as fetal bladder); (**c**) Comparison of transcriptional expression of other urothelial marker genes (*UPIb*, *UPIIIa*, *CK20*, *CLDN5* and *FOXA1*) between undifferentiated hESCs, DE and BUCs. Relative gene expressions were normalized to *GAPDH*, and fold changes are shown as mean ± SEM (*n* = 3, * *p* < 0.05); (**B**) Comparison of transcriptional activation levels of (**a**) *UPII* and (**b**) *CK7* at various concentrations of RA (0.1 to 20 μM). Untreated sample was negative control. Expression levels of transcripts were normalized to GAPDH, and fold changes are shown as mean ± SEM (*n* = 3, * *p* < 0.05); (**C**) Immunocytochemistry for the expression of the key urothelial markers in hESC-derived BUCs. Expression patterns of (**a**) UPII (green); (**b**) CK8/18 (red) and (**c**) both these markers in BUCs. (**d**–**f**) Determination of superficial and basal urothelial markers expression: (**d**) superficial urothleium marker UPII (green, yellow arrow head), (**e**) basal urothelium marker P63 (red, white arrow head); and (**f**) both these markers in BUCs. Scale bar = 100 μm; (**D**) Immunocytochemistry for the expression of gap junction molecules E-cadherin (green) and ZO-1 (red) in (**a**–**c**) DE cells and (**d**–**f**) BUCs. SOX17 (white) is shown as DE marker and yellow arrow heads indicate SOX17-positive DE cells. White dashed lines designate gap junction molecules-positive regions in BUCs. Scale bar = 100 μm; (**E**) *In vitro* permeability analysis of hESC-derived DE and BUCs. (**a**) Schematic illustration of this assay; (**b**) Monolayer staining and (**c**) FITC-dextran permeability testing. Fluorescence intensities were quantified (one second counting time) and shown as mean ± SEM (*n* = 3, *** *p* < 0.001). The no monolayer sample is a negative control. Scale bar = 200 μm; (**F**) Transcriptional activation of (**a**) *OCT4* (pluripotent markers); (**b**) smooth muscle (*CALPONIN* and *MYF5*); (**c**) Intestine (*CDX2* and *FABP2*); and (**d**) liver (*AFP* and *ALB*) were evaluated by q-PCR in hESC-derived bladder urothelium. Relative expression values were normalized to *GAPDH*, and fold-changes are shown by mean ± SEM (*n* = 3).

To determine optimal conditions for differentiation into BUCs, hESC-derived DE cells were cultivated in K-SFM supplemented with 10 μM RA for 12 days. Transcriptional expression of urothelium-specific genes *UROPLAKINII* (*UPII*) and *CYTOKERATIN7* (*CK7*) was significantly enhanced after induction day 3, as compared to induction day 0 (Figure 3A(a,b)). Transcriptional activation of other marker genes, including *UPIIIa*, *CK20*, *CLDN5* (*Claudin 5*) and *FOXA1* were notably activated in BUCs, not in hESCs and DE cells (Figure 3A(c)). Of note, gene expression of *UPII* and *CK7* was significantly up-regulated by RA treatments with higher concentration more than 5 μM (Figure 3B(a,b)). Immunocytochemistry showed that UPII and CK8/18, key urothelium markers, were notably detected in BUCs (Figure 3C(a–c)). We further examined which cell types of urothelium are generated within a whole population of BUCs. Two cell types were exclusively detected after BUCs induction, UPII-positive cells (umbrella urothelium) and P63-positive cells (basal urothelium) (Figure 3C(d–f)). As shown in Figure 3D, BUCs expressed tight junction molecules E-CADHERIN and ZO-1 as functional units of barrier, whereas SOX17-positive DE cells did not express these tight junction molecules. To examine the physiologic performance of these epithelial barriers, we performed an *in vitro* permeability assay (Figure 3E(a)). Following complete monolayer formation shown by cell staining (Figure 3E(b)), BUCs exhibited lower FITC-dextran permeability compared to DE cells (Figure 3E(c)). No monolayer sample was a negative control and demonstrated high permeability in the absence of an occlusive BUCs monolayer (Figure 3E(c)). To examine the effect of the extracellular matrix (ECM) on differentiation of BUCs, we compared four different ECM: matrigel (MG), fibronectin (FN), collagen type I (Col1) and gelatin. After BUCs induction, hESC-derivatives showed significantly higher transcriptional expression of *UPII* and *CK7* when cultured on MG, compared to other ECM (Figure S1A).

To determine whether our differentiation condition induces hESCs into non-urothelial lineage cells, we evaluated transcriptional expression of marker genes of ESCs (undifferentiated cells), smooth muscle cells (other structural units of bladder), and other endoderm lineage cells (intestine and liver cells). Transcripts of the pluripotent marker genes, including *OCT4* and *NANOG*, were markedly reduced compared to undifferentiated hESCs (Figure 3F(a)). Expression levels of smooth muscle marker genes, *ACTA2*, *CALPONIN* and *MYF5*, were not up-regulated in BUCs compared to human fetal bladder (Figure 3F(b)). Marker genes of intestine (*CDX2* and *FABP2*) and liver (*AFP* and *ALB*) were also not transcriptionally activated (Figure 3F(c,d)). We further examined transcriptional expression of marker genes of mesodermal and ectodermal origin cells. Transcripts of *RUNX2* (bone), *PECAM1* and *TIE2* (vascular endothelium), *PAX2* (kidney), and *TUJ1* and *MAP2* (neuron) were not detectable in hESC-derived BUCs (Figure S2). These results indicate that the differentiation system in the present study can efficiently specify hESCs into BUCs, not other lineage cells.

#### 2.1.3. Sequential Differentiation of hiPSCs into DE and BUCs

**Figure 4.**
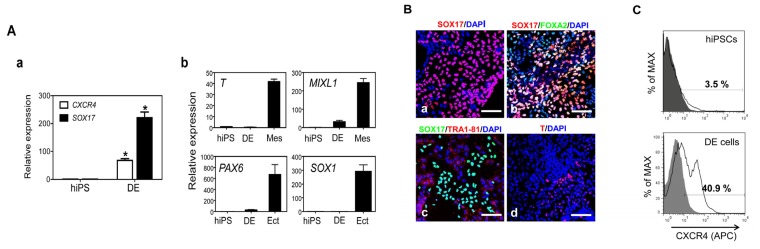
hiPSCs differentiate into DE cells. (**A**) (**a**) Transcriptional expression of the DE marker genes *CXCR4* and *SOX17* in hiPSC-derived DE cells compared to undifferentiated hiPSCs. Relative expression of marker genes was normalized to *GAPDH* and fold changes were shown as mean ± SEM (*n* = 3, * *p* < 0.05); (**b**) Determination of differentiation toward other germ layers, such as mesoderm and ectoderm, in the population of DE-induced cells. Expression of mesoderm (*T* and *MIXL1*) and ectoderm (*PAX6* and *SOX1*) marker genes was evaluated by q-PCR. hiPSCs (designated as hiPS) was a negative control. Mesodermal (Mes) and ectodermal cells (Ect) were positive controls; (**B**) Immunofluorescence of (**a**,**b**) DE markers (SOX17 and FOXA2); an (**c**) undifferentiated marker (TRA1-81); and a (**d**) mesodermal marker (T) in the DE cells. Scale bar = 200 μm; (**C**) Flow cytometry analysis for expression of the DE marker CXCR4 in hiPSCs and hiPSC-derived DE cells.

We next investigated whether hiPSCs can differentiate into bladder urothelium under the same differentiation condition as hESCs. Similar to hESCs, transcripts of *SOX17* and *CXCR4* were enriched at day five after treatment with AW (Figure 4A(a)). Genes specific to other lineages were not activated after DE induction from hiPSCs (Figure 4A(b)). The key DE marker SOX17 was markedly detected in the nuclei of hiPSC-derived DE cells by immunocytochemistry (Figure 4B(a)). SOX17 and FOXA2 were co-expressed in the nuclei of DE cells (Figure 4B(b)). However, SOX17-expressing cells did not express a pluripotent marker TRA1-81 and vice versa (Figure 4B(c)). In addition, T-expressing cells were hardly detectable in hiPSC-derivatives after DE induction (Figure 4B(d)). Finally, flow cytometry analysis showed that approximately 40% of hiPSC-derivatives after DE induction expressed CXCR4 (Figure 4C).

**Figure 5.**
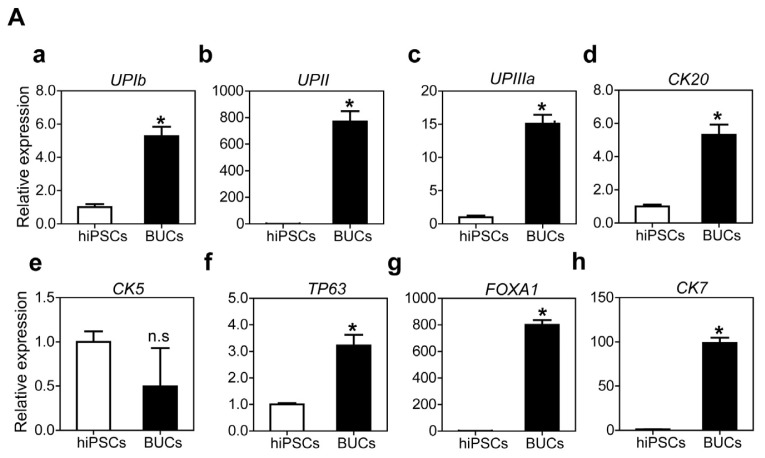

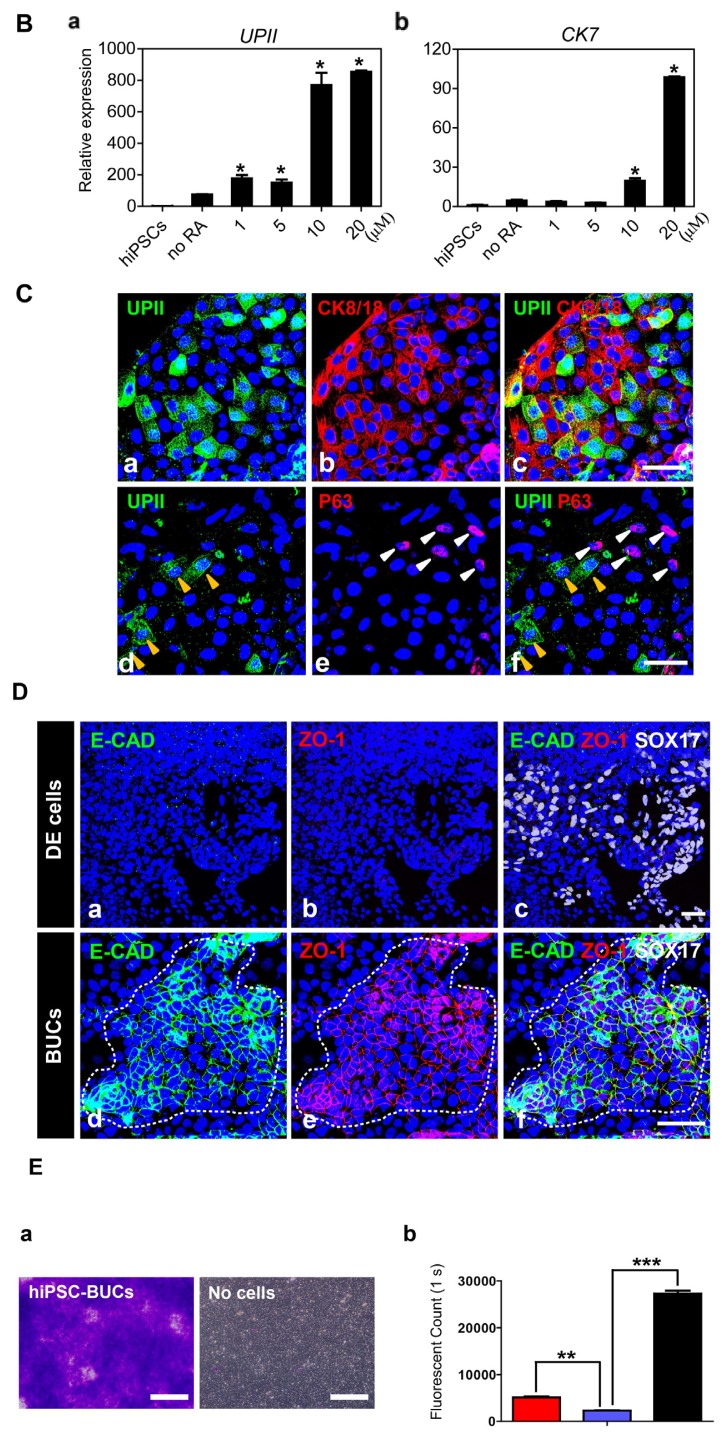

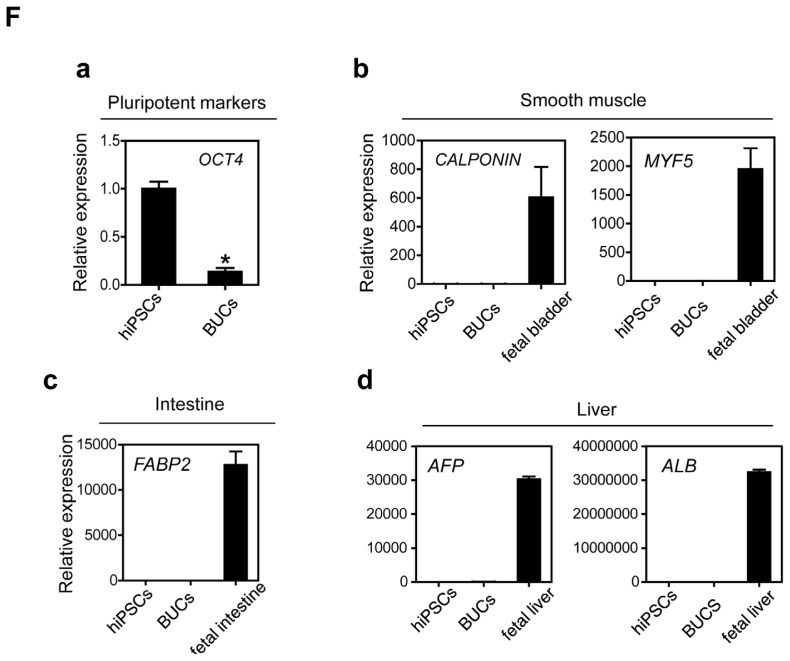
Specification of BUCs from hiPSC-derived DE cells. (**A**) Comparison of transcriptional expression of urothelial marker genes (**a**, *UPIb*; **b**, *UPII*; **c**, *UPIIIa*; **d**, *CK20*; **e**, *CK5*; **f**, *TP63*; **g**, *FOXA1*; **h**, *CK7*) between undifferentiated hiPSCs and hiPSC-derived urothelium. Relative gene expressions were normalized to *GAPDH*, and fold changes were shown as mean ± SEM (*n* = 3; * *p* < 0.05); (**B**) Comparison of transcriptional expression levels of (**a**) *UPII* and (**b**) *CK7* at various concentrations of RA (0.1 to 20 μM). Untreated sample was a negative control. Expression levels of transcripts were normalized to *GAPDH*, and fold changes were shown as mean ± SEM (*n* = 3; * *p* < 0.05); (**C**) Immunocytochemistry for the expression of the key urothelial markers in hiPSC-derived BUCs. Expression patterns of (**a**) UPII (green); (**b**) CK8/18 (red) and (**c**) both these markers; (**d**–**f**) Evaluation of superficial and basal urothelial markers expression: (**d**) superficial urothleium marker UPII (green, yellow arrow head), (**e**) basal urothelium marker P63 (red, white arrow head), and (**f**) both these markers. Scale bar = 100 μm; (**D**) Immunocytochemistry for the expression of gap junction molecules E-cadherin (green) and ZO-1 (red) in (**a**–**c**) DE cells and (**d**–**f**) BUCs. SOX17 (white) is shown as DE marker. White dashed lines indicate the regions of gap junction molecules-expressing BUCs; Scale bar = 100 μm; (**E**) *In vitro* permeability testing of hiPSC-derived DE and BUCs. (**a**) Monolayer staining and (**b**) FITC-dextran permeability analysis. Fluorescence intensities were quantified (one second counting time) and shown as mean ± SEM (*n* = 3, ** *p* < 0.01, *** *p* < 0.001). The no monolayer sample is a negative control. Scale bar = 200 μm; (**F**) Transcriptional expression of (**a**) a pluripotency gene (*OCT4*); (**b**) smooth muscle marker genes (*CALPONIN* and *MYF5*); (**c**) an intestine maker gene (*FABP2*); and (**d**) liver marker genes (*AFP* and *ALB*) were evaluated in hiPSC-derived BUCs by q-PCR. Relative expression values were normalized to *GAPDH*, and fold-changes were shown by mean ± SEM (*n* = 3, * *p* < 0.05).

For further differentiation into BUCs, DE cells were cultured in K-SFM supplemented with 10 μM RA for six days. Transcriptional activation of umbrella urothelium (*UPIb*, *UPII*, *UPIIIa*, *CK20*) and general urothelial marker genes (*FOXA1*, *CK7*) were significantly activated in hiPSC-derived BUCs compared to undifferentiated hiPSCs (Figure 5A(a–d,g,h), respectively). However, transcripts of *P63* and *CK5*, basal urothelium marker genes, slightly increased or were not enhanced in hiPSC-derived BUCs (Figure 5A(e,f)). Additionally, immunocytochemistry showed that hiPSC-derived BUCs expressed the key urothelium markers UPII, CK8/18 and P63 (Figure 5C) as well as tight junction proteins E-CADHERIN and ZO-1 (Figure 5D). Similar to hESCs, hiPSCs-derived BUCs exhibited lower permeability compared to hiPSCs-derived DE cells in a FITC-dextran permeability testing (Figure 5E). Transcriptional activation of *UPII* and *CK7* were significantly enhanced after BUCs induction in hiPSC-derivatives when cultured on MG, compared to other ECM (Figure S1B). Finally, we confirmed that hiPSC-derived BUCs did not express pluripotent (*OCT4*), smooth muscle (*CALPONIN* and *MYF5*), intestine (*FABP2*) and liver marker genes (*AFP* and *ALB*) (Figure 5F). Additionally, transcription of other lineage-specific genes, including bone (*RUNX2*), vascular endothelium (*PECAM1* and *TIE2*), kidney (*PAX2*), and neuron (*TUJ1* and *MAP2*) were not activated in hiPSC-derived BUCs (Figure S2). These results indicate that hiPSCs can also differentiate in a step-wise fashion into bladder urothelium under a serum- and feeder-free condition using RA treatment.

### 2.2. Discussion

Typically, urothelium derivation from human stem cells is performed either by co-culturing on bladder stromal cells or by culturing in a conditioned medium containing animal-serum [18,20–22]. Here, we showed for the first time that hPSCs can differentiate into BUCs under a chemically defined serum- and feeder-free system. Our protocol without animal serum and feeder cells can improve the clinical safety of hPSC-derived BUCs. Although animal models for studying the development of urogenital tracts have been well established for decades [23–25], direct translation of key findings to human is limited due to species differences. Thus, this system will provide new insights in the study of human urothelial development and disease modeling.

To establish a reliable platform for the differentiation of hPSCs into BUCs, we applied a developmental stage-dependent approach recapitulating *in vivo* organ development. Urothelium from different regions of the lower urinary tract differs in developmental origin [26]. The urothelium lining the bladder wall and urethra are derived from DE, while urothelium lining the renal pelvis, ureters and bladder trigone are derived from mesoderm [27,28]. Thus, we first differentiated hPSCs into DE, based on our hypothesis that hPSC-derived DE cells can be further efficiently specified into BUCs. Interestingly, DE induction efficiency was slightly different between hESCs and hiPSCs. This difference can be attributed to different epigenetic signatures of hESCs and hiPSCs that make reprogrammed hiPSCs more resistant to signaling stimulation for differentiation [29,30].

For further specification of hPSC-derived DE into BUCs, we used keratinocyte-specific serum free medium (K-SFM) as a basal differentiation medium, as well as retinoic acid (RA) as a key exogenous factor. K-SFM supplemented with bovine pituitary extract and recombinant epidermal growth factor is widely used for *in vitro* human urothelial cell culture [31–33]. In addition, mouse ESCs (mESCs) were able to differentiate into urothelial lineage cells expressing *uroplakin 2* when cultured in a specific keratinocyte medium [17]. RA is critical in the regional patterning and specification of endodermal lineage cells along the rostro-caudal axis [34], and is a potent regulator of the distinguishable heterogeneity of bladder urothelial keratinization [35]. The fate mapping study found marked up-regulation of RA signaling in both embryonic urothelium and adult regenerating urothelium, and directed embryonic progenitor cells specifying into urothelial cells *in vivo* [36]. In addition, RA treatment effectively induced urothelial lineage cells derived from mESCs by regulating the GATA4/6 signaling pathway *in vitro* [15]. Similar to these observations, RA treatment efficiently directs hPSC-derived DE cells toward BUCs in a dose dependent manner in this study. These BUCs significantly expressed several marker genes of urothelium such as UROPLAKIN and CYTOKERATIN, but did not express marker genes of other endoderm (liver and intestine), mesoderm and ectodermal linages cells. In addition, BUCs expressed tight junction proteins (E-CADHERIN and ZO-1) and demonstrated low FITC-dextran permeability as their physiologic performance in a permeability testing, indicating they possessed the functional units of barrier on their surfaces.

Although we differentiated hPSCs into BUCs under a chemically defined system with detailed phenotypic characterization, there are several limitations to the present study. First, our results showed only about 40% induction efficiency of CXCR4-positive DE cells. To improve induction efficiency, we can examine the effects of other growth factors and differentiation medium on hPSC differentiation into DE and BUCs. Alternatively, the isolation of DE cells with a proper surface marker can be helpful to obtain more homogenous population of DE. Furthermore, specific isolation and large expansion techniques for hPSC-derived BUCs should be developed before considering clinical applications. Second, our work provided a terminally differentiated monolayer of BUCs derived from hPSCs, not a layered structure consisting of basal cells, intermediate cells, and apical (or umbrella) cells. In this study, BUCs primarily expressed UPK (UPIb, UPII and UPIIIa) and CK20, whereas they weakly expressed P63 and hardly expressed CK5 at transcription and protein levels. Basal urothelium expresses CK5 and P63, intermediate urothelium expresses P63 and a certain type of UPK without CK5, and apical urothelium expresses UPK and CK20, but not CK5 or P63 [36]. Therefore, our differentiation protocol may be more suitable to induce apical urothelium rather than basal or intermediate urothelium. If BUCs are generated as terminally differentiated apical cells with monolayer structure, they will die without cell replacement. Before providing these BUCs for bladder regeneration, they should be optimized to make a layered structure with basal, intermediate and apical cells. Finally, we performed only *in vitro* experiments that cannot fully recapitulate *in vivo* functionality. For future clinical applications, *in vivo* feasibility of hPSC-derived BUCs should be determined.

## 3. Experimental Section

### 3.1. Generation of hiPSCs Derived from CRL-2097 Fibroblasts

We generated a human induced pluripotent stem cell (hiPSCs) line derived from human foreskin fibroblasts (CRL-2097) by ectopic expression of OCT4, SOX2, KLF4, and C-MYC (OSKM) as previously described [37]. After plating 1 × 10^5^ CRL-2097 cells on 35 mm^2^ dishes, we added four retroviral factors (OSKM) with 8 μg/mL polybrene. One day after viral infection, cells were washed three times with PBS, and fresh medium added. After two or three more days, cells were detached from the dish and 1 × 10^4^ of cells were plated on 6-well dishes coated with 0.1% gelatin. One day after re-plating, we removed the medium and added 2 mL of hESC medium. The hESC medium consisted of DMEM/F12 mixed with 20% Knockout™ Serum Replacement (Invitrogen, Carlsbad, CA, USA), 1% penicillin-streptomycin (PenStrep, Invitrogen, Carlsbad, CA, USA), 1% nonessential amino acids (NEAA, Invitrogen, Carlsbad, CA, USA), 2 mM l-glutamate (Invitrogen), 0.1 mM β-mercaptoethanol (Invitrogen, Carlsbad, CA, USA) and 10 ng/mL FGF2 (R&D Systems, Minneapolis, MN, USA). Two to three weeks after infection, we observed various types of colonies and picked colonies similar to hESCs morphology.

### 3.2. Maintenance of Human Pluripotent Stem Cells

The hESCs (H9, WiCell) and hiPSCs were maintained in hESC medium on mitomycin C-treated mouse embryonic fibroblasts (MEF) following methods described in our previous report [37]. Every six days, hPSC colonies were cut into small pieces of similar size and transferred into new culture dishes. We used hPSCs between passages 40 and 70 in this study.

### 3.3. Differentiation of BUCs from hPSCs

To differentiate hPSCs into DE cells, we modified and optimized the previously reported DE induction protocol [38] for our culture conditions. Undifferentiated hPSCs were transferred onto Matrigel (MG, 1:40 dilution; BD Biosciences, Bedford, MA, USA)-coated four-well culture dishes in mTeSR-1 medium (Stemcell Technologies, Vancouver, BC, Canada) supplemented with 10 ng/mL FGF2 for feeder-free culture. After four days of culture, hPSCs were incubated in serum-free DE induction medium (RPMI-1640 medium containing 2% B27, 2 mM l-glutamine, 1% PenStrep) supplemented with 100 ng/mL Activin A and 50 ng/mL Wnt3a (R&D Systems, Minneapolis, MN, USA), for five days. The DE induction medium was refreshed every two days. The DE induction medium was then replaced with K-SFM supplemented with 10 μM RA (Sigma-Aldrich, St. Louis, MO, USA) for further specification of DE cells into BUCs. The K-SFM was changed every two days. Mesodermal and ectodermal cells derived from hPSCs, as previously described [39,40], were used as lineage-positive controls.

### 3.4. RNA Preparation and Real Time RT-PCR

Total RNA was isolated from the experimental samples using Easy-Blue^®^ (Invitrogen, Carlsbad, CA, USA), followed by cDNA synthesis using oligo-dT and Moloney-murine leukemia virus reverse transcriptase (Enzynomics, Seoul, Korea). Transcriptional expression of target genes was analyzed by quantitative real time RT-PCR with SYBR^®^ Green using a Bio-Rad CFX manager (Bio-Rad Laboratories, Hercules, CA, USA). All reaction were conducted using the following protocol: 95 °C for 10 min (initiation step), followed by 40 cycles of 95 °C for 15 s (denaturation step), 60 °C for 15 s (annealing step) and 72 °C for 30 s (elongation step). Primers of the target genes were constructed using the primer design tools in NCBI (http://www.ncbi.nlm.nih.gov/tools/primer-blast), and the efficiency of each primer set was confirmed by standard curve analysis. All primers were used at a final concentration of 0.5 pmole for q-PCR. All experiments used three independent samples and each q-PCR reaction was performed in duplicate to diminish technical errors. For relative quantification, transcriptional expression of target genes was normalized to glyceraldehyde-3-phosphate dehydrogenase (*GAPDH*) gene expression as an internal control. Differences in transcriptional expression between experimental and control groups were analyzed by the ΔΔ*C*t method. Representative values for transcriptional expression of target genes are indicated as fold changes compared to negative controls. The sequences of the primers used in this study are described in Table S1.

### 3.5. Immunocytochemistry

Samples were washed with PBS and fixed in 4% formaldehyde (Sigma-Aldrich, St. Louis, MO, USA) for 30 min at room temperature (RT). After washing three times with PBS containing 0.1% Tween-20 (PBST), cells were permeabilized by treatment with 0.3% Triton X-100 (Sigma-Aldrich, St. Louis, MO, USA) for 20 min. The samples were then blocked with 5% donkey serum (Jackson ImmunoResearch Laboratories, West Grove, PA, USA) for one hour at RT. Samples were incubated with primary antibodies at 4 °C overnight. After rinsing three times with PBST, the samples were incubated with secondary antibody for one hour at RT. After washing four times with PBST, the samples were incubated in VECTASHIELD Mounting Medium containing DAPI (1:1000, Vector Laboratories, Burlingame, CA, USA) for 5 min, followed by additional rinsing twice with PBST. The samples were examined using a Zeiss LSM 510 confocal microscope (Carl Zeiss, Oberkochen, Germany). The primary antibodies used in this study are described in Table S2.

### 3.6. Flow Cytometric Analysis

Cells were treated with Acutase (Innovative Cell Technologies, San Diego, CA, USA) at 37 °C for 10 min. Dissociated cells were rinsed twice with FACS buffer (PBS containing 5% fetal bovine serum) by centrifugation at 300× *g* for 5 min. The cells were labeled with fluorophore-conjugated antibodies on ice for 30 min. Finally, the cells were rinsed twice with FACS buffer by centrifugation at 300× *g* for 5 min, and assayed using a FACSCalibur system (Becton Dickson, San Jose, CA, USA) according to the manufacturer’s instructions. Data were analyzed using the FlowJo software, version 7.2.5 (Tree Star, Inc., Ashland, OR, USA). Phycobiliproteins (PE)-conjugated mouse IgG isotype control (1:100, BD Biosciences, Bedford, MA, USA), PE-mouse anti human SSEA4 (1:100, BD Biosciences, Bedford, MA, USA), allophycocyanin (APC)-conjugated mouse IgG isotype control (1:100, BD Biosciences, Bedford, MA, USA), and APC-mouse anti human CXCR4 antibody (1:100, BD Biosciences, Bedford, MA, USA) were used in this study.

### 3.7. In Vitro Permeability Assay

To examine the functional performance of BUCs barriers, we used the Millipore *In Vitro* Vascular Permeability Assay Kit (Merk Millipore, Billerica, MA, USA). Cells were seeded onto the collagen-coated inserts (2 × 10^5^ cells/mL) and cultured until complete monolayer formation occurred. After the medium were carefully removed from the inserts without disturbing the cell monolayer, 150 μL of FITC-dextran working solution (1:40) were added to each insert. Samples were incubated for 20 min, protected from light, at RT. Permeation of FITC-dextran was stopped by removing the inserts from the wells. The medium containing FITC-dextran that passed monolayer was thoroughly mixed and 100 μL of the medium transferred into wells of black 96-well plate. Fluorescence intensities were measured on a multi-well microplate reader (PerkinElmer, Waltham, MA, USA) with filters appropriate for 485 and 535 nm excitation and emission, respectively. After completion of permeability assay, the cells monolayer was stained for determining the monolayer integrity. The medium was carefully removed from the inserts and 100 μL of cell stain solution was added. The inserts were incubated for 20 min at RT and washed twice with PBS. The inserts were left in the second rinse with PBS and the monolayer integrity was observed with a stereomicroscope.

### 3.8. Statistical Analysis

All experiments used three independent samples for statistical analysis in this study. All data are shown as the mean ± SEM. The statistical significance of experiment outcomes was calculated using either Student’s *t* test or one-way ANOVA with *post-hoc* test of multiple comparisons. Null hypotheses of no difference were rejected if *p*-values were less than 0.05. We performed all statistical analysis using GraphPad Prism 5.0 (GraphPad Software, Inc., La Jolla, CA, USA).

## 4. Conclusions

In summary, hPSCs efficiently differentiated into BUCs under a chemically defined system using developmental stage-dependent approach. These hPSC-derived BUCs will be promising cell sources for bioengineered bladder, as well as providing an *in vitro* platform for studying normal and pathological development of the human bladder urothelium.

## Supplementary Information



## Abbreviations

hPSCs: Human pluripotent stem cells
hESCs: Human embryonic stem cells
hiPSCs: Human induced pluripotent stem cells
DE: Definitive endoderm
BUCs: Bladder urothelial cells
*UP*: UROPLAKIN
*CK*: CYTOKERATIN
AW: Activin and Wnt3a
RA: Retinoic acid
K-SFM: Keratinocyte-specific serum-free medium
