# Supplementary material for: Generation of Bladder Urothelium from Human Pluripotent Stem Cells under Chemically Defined Serum- and Feeder-Free System
Source: Int J Mol Sci. 2014 Apr 25;15(5):7139–57. doi: 10.3390/ijms15057139 (PMC4057664; doi:10.3390/ijms15057139)

Supplementary Information

Table S1. The information of real time RT-PCR primers.

Gene	Forward sequence	Reverse sequence
GAPDH	ctt cgc tct ctg ctc ctc ct	gtt aaa agc agc cct ggt ga
T	cag tgg cag tct cag gtt aag aag ga	cgc tac tgc agg tgt gag caa
MIXL1	tcc agg atc cag gta tgg tt	cgt ttc agt tcc agg agc ac
EOMES	atg ctg aag agt ata gta aag aca	aac acc acc aag tcc atc
SOX17	aaa gac cca ggg tac cta aa	agg aag aca aat tct cac agc ag
CXCR4	ggt ggt cta tgt tgg cgt ct	tgg agt gtg aca gct tgg ag
PAX6	agc cca gta taa gcg gga gt	cta gcc agg ttg cga aga ac
SOX1	cac aac tcg gag atc agc aa	ggt act tgt aat ccg ggt gc
UPIb	ggg aca gac aag gtg cct gtt at	tat tgg ctg gct tgc ttc tct cca
UPII	cag tgc tgc ctc acc ttc caa ca	tgg taa aat ggg agg aaa gtc aa
UPIIIa	tca ctg gca ccc acg agg tct	cgt tga gcc cag tgg ggt gtt
CK5	caa ccc act agt gcc tgg tt	gac aca ctt gac tgg cga ga
CK7	tgt ggt gct gaa gaa gga tgt gga	tgt caa ctc cgt ctc att gag ggt
CK20	ctg aat aag gtc ttt gat gac c	atg ctt gtg tag gcc atc ga
FOXA1	gaa gat gga agg gca tga aa	gcc tga gtt cat gtt gct ga
TP63	tgc agg act cgg acc tga gt	tgt tca gga gcc cca ggt t
CLDN5	ctg ttt cca tag gca gag cg	aag cag att ctt agc ctt cc
CDX2	gca gag caa agg aga gga aa	aag ggc tct ggg aca ctt ct
FABP2	tgc agc tca tga caa ttt ga	ccc tga gtt cag ttc cgt ct
AFP	agc ttg gtg gtg gat gaa	tct gca atg aca gcc tca ag
ALB	tgc aca gaa tcc ttg gtg aa	ttc acg agc tca aca agt gc
ACTA2	tca atg tcc cag cca tgt at	cag cac gat gcc agt tgt
CALPONIN	agg ctc cgt gaa gaa gat ca	cca cgt tca cct tgt ttc ct
MYF5	cca cct cca act gct ctg at	agg tga tcc ggt cca cta tg
RUNX2	gac agc ccc aac ttc ctg t	ccg gag ctc agc aga ata at
PECAM1	tgc gaa tcg atc agt gga	acc ggg gct atc acc ttc
TIE2	cct tag tga cat tct tcc	gca aaa atg tcc acc tgg
PAX2	acg ccc att aaa gca cag	tta cag aga aag agc caa caa a
TUJ1	ggg cct ttg gac atc tct tc	cct ccg tgt agt gac cct tg
MAP2	gtg gcg gac gtg tga aaa ttg ag	ctg gat ctg cct ggg gac tgt g

Table S2. The information of primary antibodies.

Antibody	Host	Dilution factor	Industry
T	Goat	1:200	R&D Systems, Minneapolis, MN, USA
TRA1-81	Mouse	1:200	Millipore, Billerica, MA, USA
SOX17	Goat	1:100	R&D Systems, Minneapolis, MN, USA
FOXA2	Rabbit	1:200	R&D Systems, Minneapolis, MN, USA
GATA4	Rabbit	1:200	Santa Cruz Biotechnology, Santa Cruz, CA, USA
UP II	Goat	1:100	Santa Cruz Biotechnology, Santa Cruz, CA, USA
CK8/18	Mouse	1:200	Abcam, Cambridge, MA, USA
P63	Rabbit	1:100	Cell Signaling Technology, Danvers, MA, USA
E-CADHERIN	Mouse	1:50	BD bioscience, Franklin Lakes, NJ, USA
ZO-1	Rabbit	1:300	Millipore, Billerica, MA, USA

Figure S1. Comparison of the effect of extracellular matrix (ECM) on bladder urothelial cells (BUCs) differentiation from hPSCs. Transcriptional expression levels of the key marker genes (**a**) *UPII (UROPLAKINII)* and (**b**) *CK7 (CYTOKERATIN 7)* in the (**A**) hESC- and (**B**) hiPSC-derivatives cultured on different ECM: matrigel (MG), fibronectin (FN), collagen type I (Col1), and gelatin. Undifferentiated cells are the negative controls. Relative gene expressions were normalized to *GAPDH*, and fold changes are shown as mean ± SEM (n = 3, * $p < 0.05$).

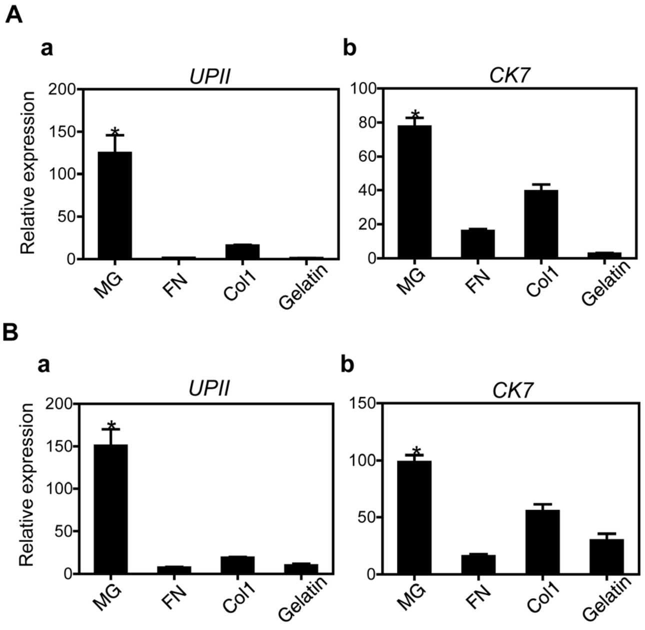

Figure S2. Evaluation of transcriptional activation of other inducible lineages markers in hPSC-derived BUCs. Transcriptional expression of (**a**) *RUNX2* (bone); (**b**) *PECAM1* and *TIE2* (vascular endothelium); (**c**) *PAX2* (kidney); and (**d**) *TUJ1* and *MAP2* (neuron) were analyzed by q-PCR in hPSC-derived BUCs. Relative expression values were normalized to GAPDH, and fold-changes are shown by mean ± SEM (n = 3). Mesodermal and ectodermal origin cells derived from hPSCs were used as lineage-positive controls.

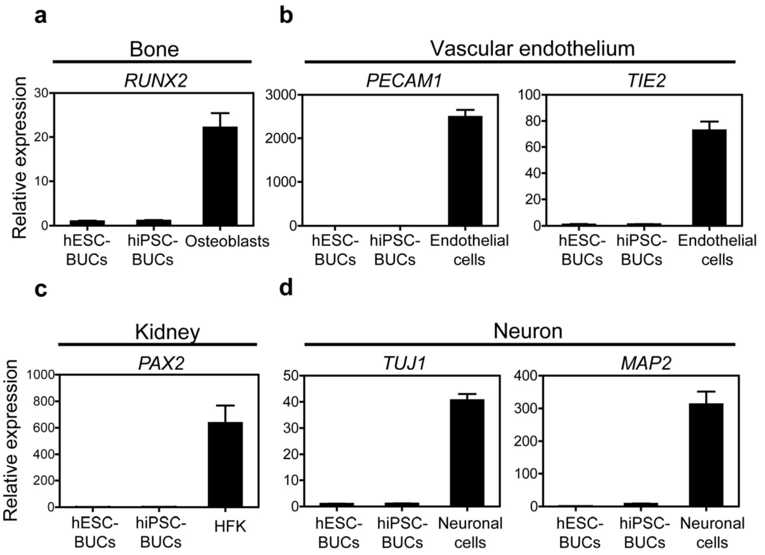

Supplement: Supplementary file 1 [file ijms-15-07139-s001.pdf]
